# Association of Urinary Iodine Concentration with Depressive Symptoms among Adults: NHANES 2007–2018

**DOI:** 10.3390/nu14194165

**Published:** 2022-10-07

**Authors:** Shumin Chen, Kaiwen Cui, Jia Luo, Dongfeng Zhang

**Affiliations:** Department of Epidemiology and Health Statistics, Qingdao University Medical College, Qingdao 266071, China

**Keywords:** urinary iodine concentration, depressive symptoms, National Health and Nutrition Examination Survey, dose-response

## Abstract

The association between iodine status and depressive symptoms has not been investigated in the general population. Therefore, we drew 8935 participants from the National Health and Nutrition Examination Survey (NHANES) 2007–2018 to explore their association. In NHANES, Inductively Coupled Plasma Dynamic Reaction Cell Mass Spectroscopy was utilized to measure urinary iodine concentration (UIC), and Patient Health Questionnaire-9 was used to assess depressive symptoms. Meanwhile, we fitted logistic regression and restricted cubic spline models. We found that high UIC was associated with a higher prevalence of depressive symptoms than the normal UIC group (OR: 1.50, 95% CI: 1.04–2.16). This association was particularly pronounced and further strengthened among females (OR: 1.90, 95% CI: 1.19–3.01) and participants aged 40–59 (OR: 1.90, 95% CI: 1.11–3.25). Moreover, we found that low UIC was associated with a high prevalence of depressive symptoms among females (OR: 1.50, 95% CI: 1.02–2.18). Moreover, the dose-response relationship between UIC and depressive symptoms presented a general trend of decreased, steady transiently, and then increased. We found that participants with high UIC had a higher prevalence of depressive symptoms than those with normal UIC. Meanwhile, we also found that females with low UIC had higher odds of reporting depressive symptoms.

## 1. Introduction

Depression is a growing public health concern linked to poor quality of life and increased mortality [1,2], affecting 5–10% of the US population [3]. The World Health Organization (WHO, Geneva, Switzerland) predicts that major depressive disorder will be the largest cause of the global disease burden by 2030 [4]. Therefore, it is critical to investigate the modifiable factors of depression.

Many studies have shown that demographic, genetic, environmental, and dietary factors were associated with depression [5,6,7,8,9,10,11]. Among them, the relationships between nutrients and depression have been widely explored. For example, a recent study showed that dietary vitamin C was negatively associated with depressive symptoms [9]. Several other studies have found that zinc, iron, copper, magnesium, and selenium were inversely associated with depressive symptoms [10,11].

As an essential micronutrient, iodine is involved in synthesizing thyroid hormones (THs) [12,13]. It has been shown that iodine can alter certain enzyme activities in the central nervous system [14,15], and THs can regulate central nervous system function relating to neuropsychiatric disorders [16,17].

Several studies have explored the relationship between iodine status and depression, with inconsistent findings. A case-control study among adolescents aged 8 to 16 found that high urinary iodine concentration strongly correlated to decreased risk of depression [18]. In contrast, a follow-up study of healthy pregnant women reported that pregnant women who took additional iodine-containing vitamin supplementation had higher depression test scores than the control group [19]. Furthermore, a cross-sectional study of patients with euthyroid nodular goiter (ENG) found that low iodine levels were associated with anxiety, but not significant associations with depression [20]. However, these studies had relatively small sample sizes and focused on adolescents, pregnant women, or ENG patients. Moreover, few studies have yet detected the dose-response relationship between iodine status and depression.

Considering that iodine is mainly eliminated by the kidneys [21] and urinary iodine concentration (UIC) is the most common indicator to assess the population’s iodine status [22], we explored the association and the dose-response relationship between UIC and depressive symptoms in the general population using the National Health and Nutrition Examination Survey (NHANES) 2007–2018.

## 2. Materials and Methods

### 2.1. Study Population

The NHANES utilizes a complex multistage probability sampling design to select the nationally representative sample, aiming to assess US non-institutionalized civilians’ health and nutritional status. The Centers for Disease Control and Prevention conducts a survey every two years [23]. All the participants signed informed consent. The Research Ethics Review Board of the National Center for Health Statistics authorized the investigation protocol (Protocol #2005-06, Protocol #2018-01) [24].

**Figure 1:**
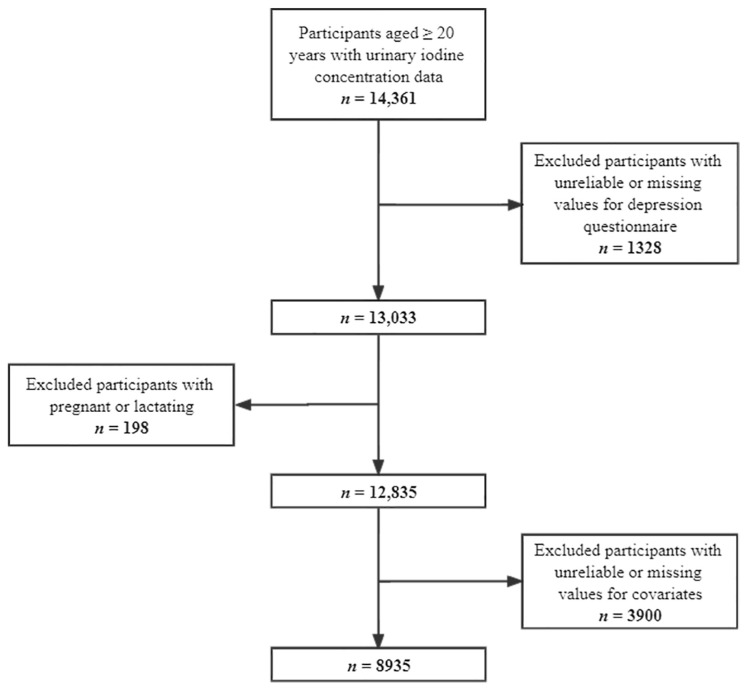
Flow chart of the screening process for the selection of eligible participants.

We obtained publicly available data for 14,361 participants with UIC data aged ≥ 20 years from the 2007–2018 survey cycles. Then, we excluded participants with unreliable or missing values for the depression questionnaire (*n* = 1328). Moreover, we removed pregnant or lactating females (*n* = 198) and individuals with unreliable or missing values for covariates (*n* = 3900). Overall, our study included 8935 participants (4717 males and 4218 females). Figure 1 depicts the screening process for qualified individuals in detail.

### 2.2. Urinary Iodine Concentration Measurement

In NHANES, the Inductively Coupled Plasma Dynamic Reaction Cell Mass Spectroscopy was utilized to measure UIC in spot urine samples. The laboratory’s equipment, procedures, or location did not change during the six cycles. On the NHANES website [25], detailed instructions about collecting and processing samples can be found. 

### 2.3. Depressive Symptoms Assessment

The Patient Health Questionnaire-9 (PHQ-9) was used to assess depressive symptoms, a simple and validated self-rating scale for how often depressive symptoms have appeared over the past two weeks [26]. It consists of nine items, and each question is scored from 0 to 3. The total score ranges from 0 to 27, with higher scores corresponding to more significant depressive symptoms. Our study defined having depressive symptoms as participants with a score ≥10 [27].

### 2.4. Covariates

In the current study, we adjusted age, sex, race, education level, annual household income, body mass index (BMI), marital status, physical activity, caffeine intake, total energy intake, sodium intake, smoking status, alcohol consumption, urinary creatinine levels, hypertension, diabetes, and stroke history for controlling the confounding bias, based on previous research [28,29,30,31]. Table S1 shows the details on covariables.

### 2.5. Statistical Analysis

We constructed a 12-year sample weight to ensure the study sample represents the non-institutionalized civilian population, as recommended by the NHANES [32]. According to the epidemiological criteria for iodine nutrition proposed by the WHO [22], we divided the participants into four groups according to UIC (μg/L) as follows: low UIC, <100 µg/L; normal UIC, 100–199 µg/L; slightly high UIC, 200–299 µg/L; and high UIC, ≥300 µg/L. We selected the normal UIC group as the reference group. 

We performed the Kolmogorov–Smirnov method to test normality for quantitative variables. Then, we used Student’s *t*-test to examine participants’ characteristics between with and without depressive symptom groups for normally distributed continuous data, the Mann–Whitney U test for continuous skewed data, and the Chi-square test for categorical variables.

We used logistic regression models to examine the correlation between UIC and depressive symptoms. Model 1 did not adjust any covariate, model 2 adjusted for age and sex, and model 3 incorporated all covariates. Given that the prevalence of depression varies by age and sex [33], we conducted stratified analyses by age and sex. Moreover, we investigated their dose-response relationship using restricted cubic spline models with three knots (50 µg/L, 150 µg/L, 350 µg/L) in model 3. Finally, we conducted multiple sensitivity studies to check whether our findings were reliable. First, we minimized the confounding by excluding participants using amiodarone, thyroid hormone replacement agents, or anti-thyroid agents and subjects with severe renal dysfunction. Second, we explored the effect of unmeasured or unknown confounding factors between UIC and depressive symptoms by calculating the E-value [34]. Our study used Stata 15.0 to conduct statistical analyses and regarded a two-sided *p* ≤ 0.05 as statistically significant.

## 3. Results

Our study included 8935 participants, and the prevalence of depressive symptoms was 9.26%. Table 1 displays the baseline characteristics of the study population. Participants with depressive symptoms were more likely to be 40–59 years old, female, obese, smokers, drinkers, live alone, and have hypertension, diabetes, and stroke history. They were also more likely to have less education, lower household income, and less physical activity. Additionally, participants with depressive symptoms had higher UIC than those without depressive symptoms.

Table 2 presents the results of the logistic regression analysis. In the crude model, high UIC was related to a higher prevalence of depressive symptoms compared with the normal UIC group, and the weighted odds ratio (OR) with a 95% confidence interval (CI) was 1.55 (1.11–2.17). These results did not materially alter after adjusting for age and sex, with a weighted OR (95% CI) of 1.66 (1.18–2.33). In the multivariate-adjusted model, high UIC was still positively correlated with depressive symptoms (OR: 1.50, 95% CI: 1.04–2.16). However, the low UIC group was not significantly related to depressive symptoms, although the weighted OR in the low UIC group was above 1. 

Table 3 shows the results of stratified analysis by sex. Among females, participants in the low and high UIC groups had a higher prevalence of depressive symptoms than participants in the normal UIC group, and the weighted ORs (95% CIs) were 1.50 (1.02–2.18) and 1.90 (1.19–3.01), respectively. However, we did not observe statistical significance in males. Table 4 shows the results of stratified analysis by age. Among participants aged 40–59, high UIC was associated with a high prevalence of depressive symptoms, with a weighted OR (95% CI) of 1.90 (1.11–3.25). In other age groups, we did not find any statistical associations.

**Figure 2:**
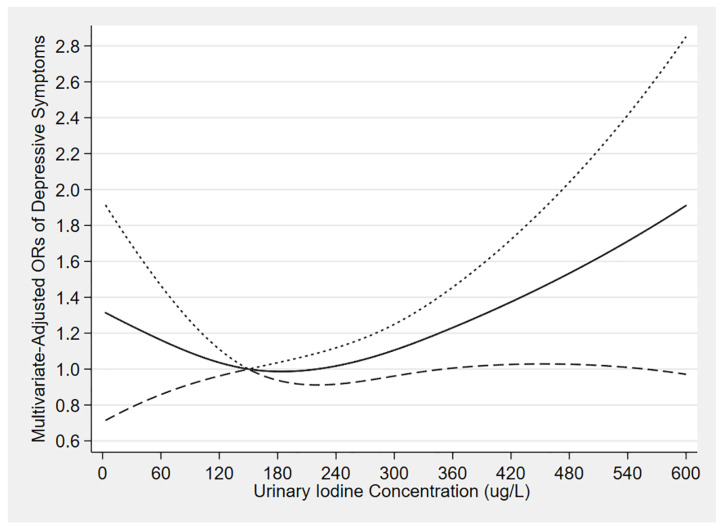
The dose-response relationship between urinary iodine concentration and the risk of depressive symptoms. Adjusted for age, sex, race, education level, annual household income, BMI, marital status, physical activity, caffeine intake, total energy intake, sodium intake, urinary creatinine level, smoking status, drinking status, hypertension, diabetes, and stroke history. Reference is median of the normal UIC group, i.e., 150 μg/L. The solid line and dash line represent the estimated odds ratios (ORs) and their 95% confidence intervals, respectively.

Figure 2 depicts the results of restricted cubic spline models. With the increase of UIC, the multivariate-adjusted ORs of depressive symptoms presented a general trend of first decreased, then steady transiently, and finally increased. Meanwhile, the relationship demonstrated statistical significance when the UIC was above 360 µg/L approximately (OR: 1.23, 95% CI: 1.01–1.46) (Table S2).

Table S3 shows the results of the sensitivity analysis. After excluding participants using amiodarone, thyroid hormone replacement agents, or anti-thyroid agents and subjects with severe renal dysfunction, the association between high UIC and high prevalence of depressive symptoms was strengthened slightly, with a weighted OR (95% CI) of 1.53 (1.00–2.34). In addition, the E-value of the high UIC group was 2.43. This value indicated that our findings were robust unless an unmeasured or unknown confounding factor has a relative risk greater than 2.43 with both UIC and depressive symptoms.

## 4. Discussion

In this cross-sectional study of 8935 participants, we found that individuals with high UIC had greater odds of reporting depressive symptoms than those with normal UIC. This association was particularly pronounced and further strengthened among females and participants aged 40–59. Moreover, we found that low UIC was related to a significant prevalence of depressive symptoms among females. The multiple sensitivity analyses suggested that our results were robust. Finally, the multivariate-adjusted ORs of depressive symptoms showed a first decreased, then steady transiently, and finally increased trend, with the increase of UIC, similar to our regression analysis results.

Some studies have explored the link between UIC and depression. Consistent with our findings, a follow-up study of pregnant women in Shenyang, Liaoning Province of China, showed that those who took additional iodine-containing vitamin supplementation had higher depression test scores than the control group [19]. Moreover, a study with a case-control design performed on Chinese adolescents aged 8 to 16 found that low but not high UIC was closely correlated to a higher risk of depression [18]. We found a similar relationship among females in the present study. However, a cross-sectional study of 102 patients with ENG found that iodine levels were negatively associated with the prevalence of anxiety, but no significant associations were found with depression [20]. In the current study, we observed that UIC was related to depressive symptoms, which is at odds with the above study. The inconsistency is likely attributable to differences in the outcome variable’s definition, study populations, and sample sizes. 

So far, the mechanisms underlying the association between excessive iodine intake and depressive symptoms have not been established but might be explained by the following aspects. Firstly, excess iodine can inhibit superoxide dismutase and glutathione peroxidase activity [35], affecting the body’s antioxidant capacity [15]. Secondly, excess iodine can reduce nitric oxide synthesis [35], affecting information transmission in the central nervous system [14,36]. Moreover, excess iodine can reduce hydrogen peroxide production and prevent thyroid peroxidase from catalyzing the organic process of iodine [37,38]. Finally, excess iodine can inhibit type II iodothyronine 5′-deiodinase activity, affecting the conversion process from T4 to T3 in the central nervous system [39]. Noteworthily, we found that iodine deficiency was related to the high risk of depressive symptoms among females. One likely reason is that iodine deficiency can cause hypothyroidism [40], which impairs normal neurogenesis and reduces the number of newborn neuroblasts and immature neurons, leading to mood disorders [41]. However, our hypotheses need future studies to confirm.

The advantages of this study are as follows. First, to our knowledge, the current study is the first to show a connection between UIC and depressive symptoms in the general population. Second, we appraised the dose-response relationship between UIC and depressive symptoms. Third, we utilized the nationally representative general population as study subjects, and the sample size is large. However, the following limitations are noteworthy. First, the cross-sectional design of our study prevented us from determining the causation between UIC and depressive symptoms. Second, the mothers of partial participants aged 40–59 might have very high levels of dioxin-like polychlorinated biphenyls during pregnancy, which might have deleterious effects on the thyroid function of the fetus and newborns [42,43]. A study noted that early exposure to endocrine environmental disruptors might influence later life health through epigenetic mechanisms [44], so these babies as adults might be more sensitive to iodine status. However, relevant data are not available, which might have influenced our results. Third, although we adjusted potential confounding factors as much as possible, it remained impossible to rule out all confounders. However, we used the E-value to evaluate the robustness of our observed association, and we discovered that unmeasured or unknown confounders were not likely to overturn the observed relationships. Fourth, our study used a single spot urine measurement to evaluate the population’s iodine status, which may be influenced by recent liquid intake [45]. However, we adjusted the urinary creatinine levels to control the effect of liquid intake as much as possible [46]. Fifth, the PHQ-9 scale is a self-assessment scale and not a clinical diagnostic standard for depression, which might mean that the results are biased.

## 5. Conclusions

In summary, our findings indicated that participants with high UIC had a higher prevalence of depressive symptoms than those with normal UIC. Meanwhile, we also found that females with low UIC had higher odds of having depressive symptoms. These remind us to pay more attention to the optimal intake of iodine. Meanwhile, it is necessary to enhance population iodine intake monitoring to evaluate the potential long-term health effects of excessive iodine exposure. Prospective cohort and trial studies should be conducted to confirm our results in the future.

## Figures and Tables

**Table 1:** Baseline characteristics of participants, NHANES 2007–2018 (*N* = 8935).

	Non-Depressive Symptoms (PHQ < 10)	Depressive Symptoms (PHQ ≥ 10)	*p* Value
Number of participants (%) ^a^	8108 (90.74)	827 (9.26)	
Age (years), *n* (%) ^a^			<0.01
20–39 years	2554 (33.95)	267 (32.74)	
40–59 years	2695 (38.70)	342 (46.21)	
≥60 years	2859 (27.35)	218 (21.05)	
Sex, *n* (%) ^a^			<0.001
Male	4391 (51.78)	326 (37.08)	
Female	3717 (48.22)	501 (62.92)	
Race, *n* (%) ^a^			<0.001
Mexican American	1140 (7.25)	115 (7.73)	
Other Hispanic	777 (4.85)	93 (5.85)	
Non-Hispanic White	3858 (71.78)	392 (64.11)	
Non-Hispanic Black	1672 (9.88)	169 (12.64)	
Other races	661 (6.24)	58 (9.66)	
Educational level, *n* (%) ^a^			<0.001
<High school	1688 (12.20)	283 (22.83)	
High school	1933 (23.39)	208 (29.73)	
>High school	4487 (64.41)	336 (47.43)	
Annual household income, *n* (%) ^a^			<0.001
<$20,000	1473 (11.23)	319 (27.68)	
≥$20,000	6635 (88.77)	508 (72.32)	
Body mass index, *n* (%) ^a^			<0.01
<25 kg/m^2^	2231 (28.07)	194 (23.00)	
25 to <30 kg/m^2^	2727 (33.00)	235 (28.29)	
≥30 kg/m^2^	3150 (38.93)	398 (48.71)	
Marital status, *n* (%) ^a^			<0.001
Not living alone	5030 (65.30)	380 (49.00)	
Living alone	3078 (34.70)	447 (51.00)	
Physical activity, *n* (%) ^a^			<0.001
Less	3060 (33.02)	420 (48.33)	
Normal	5048 (66.98)	407 (51.67)	
Smoke at least 100 cigarettes in life, *n* (%) ^a^	4025 (47.03)	535 (66.57)	<0.001
Had at least 12 alcohols drink a year, *n* (%) ^a^	1270 (14.28)	239 (31.57)	<0.001
Hypertension, *n* (%) ^a^	4427 (50.40)	494 (58.01)	<0.05
Diabetes, *n* (%) ^a^	1450 (13.67)	199 (19.77)	<0.01
Ever told you had a stroke, *n* (%) ^a^	278 (2.21)	65 (6.10)	<0.001
Caffeine intake (mg/d), median (IQR) ^b^	109.50 (180.25)	116.00 (208.00)	0.210
Total energy intake (kcal/d), median (IQR) ^b^	1940.50 (1005.50)	1836.50 (1077.50)	<0.001
Sodium intake (mg/d), median (IQR) ^b^	3159.75 (1778.75)	2905.00 (1804.5)	<0.001
Urinary creatinine level (mg/dL), median (IQR) ^b^	107.00 (102.00)	111.00 (105.00)	<0.01
Urinary iodine concentration (μg/L), median (IQR) ^b^	136.15 (162.35)	152.10 (192.60)	<0.01

Data are the number of participants (weighted percentage) or medians (interquartile ranges). PHQ-9, Patient’s Health Questionnaire-9. ^a^ Chi-square test was used to compare the percentage between participants with and without depressive symptoms. ^b^ Mann–Whitney U test was used to compare the mean values between participants with and without depressive symptoms.

**Table 2:** Weighted ORs and 95% CIs of depressive symptoms according to urinary iodine concentration.

Urinary Iodine Concentration (μg/L)	Cases/Participants	Model 1 ^a^	Model 2 ^b^	Model 3 ^c^
		OR (95% CI)	OR (95% CI)	OR (95% CI)
Low UIC (<100)	274/3170 (8.64%)	1.12 (0.84–1.52)	1.06 (0.80–1.43)	1.22 (0.91–1.65)
Normal UIC (100–199)	254/2867 (8.86%)	1.00 (reference)	1.00 (reference)	1.00 (reference)
Slightly high UIC (200–299)	116/1314 (8.83%)	1.03 (0.70–1.52)	1.10 (0.75–1.61)	1.02 (0.72–1.46)
High UIC (≥300)	183/1588 (11.55%)	1.55 (1.11–2.17) *	1.66 (1.18–2.33) **	1.50 (1.04–2.16) *

Calculated using binary logistic regression. OR, odds ratio; CI, confidence interval. ^a^ Model 1 is the unadjusted model; ^b^ Model 2 is adjusted for age and sex; ^c^ Model 3 further adjusted for race, education level, annual household income, BMI, marital status, physical activity, caffeine intake, total energy intake, sodium intake, smoking status, drinking status, hypertension, diabetes, stroke history, and urinary creatinine level. * *p* < 0.05; ** *p* < 0.01.

**Table 3:** Weighted ORs and 95% CIs of depressive symptoms stratified by sex.

Urinary Iodine Concentration (μg/L)	Cases/Participants	Model 1 ^a^	Model 2 ^b^	Model 3 ^c^
		OR (95% CI)	OR (95% CI)	OR (95% CI)
Females				
Low UIC (<100)	171/1662 (10.29%)	1.17 (0.81–1.69)	1.13 (0.79–1.64)	1.50 (1.02–2.18) *
Normal UIC (100–199)	158/1334 (11.84%)	1.00 (reference)	1.00 (reference)	1.00 (reference)
Slightly high UIC (200–299)	64/549 (11.66%)	1.32 (0.74–2.35)	1.35 (0.77–2.37)	1.15 (0.66–1.99)
High UIC (≥300)	108/669 (16.14%)	2.08 (1.38–3.15) **	2.16 (1.43–3.28) **	1.90 (1.19–3.01) **
Males				
Low UIC (<100)	103/1508 (6.83%)	1.00 (0.65–1.56)	0.99 (0.64–1.54)	0.94 (0.59–1.49)
Normal UIC (100–199)	96/1530 (6.27%)	1.00 (reference)	1.00 (reference)	1.00 (reference)
Slightly high UIC (200–299)	51/763 (6.68%)	0.81 (0.49–1.34)	0.81 (0.48–1.36)	0.81 (0.51–1.31)
High UIC (≥300)	75/914 (8.21%)	1.11 (0.69–1.77)	1.11 (0.69–1.78)	1.06 (0.64–1.75)

Calculated using binary logistic regression. OR, odds ratio; CI, confidence interval. ^a^ Model 1 is the unadjusted model; ^b^ Model 2 is adjusted for age; ^c^ Model 3 further adjusted for race, education level, annual household income, BMI, marital status, physical activity, caffeine intake, total energy intake, sodium intake, smoking status, drinking status, hypertension, diabetes, stroke history, and urinary creatinine level. * *p* < 0.05; ** *p* < 0.01.

**Table 4:** Weighted ORs and 95% CIs of depressive symptoms stratified by age.

Urinary Iodine Concentration (μg/L)	Cases/Participants	Model 1 ^a^	Model 2 ^b^	Model 3 ^c^
		OR (95% CI)	OR (95% CI)	OR (95% CI)
20–39 years				
Low UIC (<100)	88/1098 (8.01%)	0.88 (0.59–1.32)	0.86 (0.57–1.29)	0.97 (0.67–1.41)
Normal UIC (100–199)	95/939 (10.12%)	1.00 (reference)	1.00 (reference)	1.00 (reference)
Slightly high UIC (200–299)	36/359 (10.03%)	0.96 (0.57–1.60)	1.00 (0.60–1.67)	0.87 (0.50–1.51)
High UIC (≥300)	47/423 (11.11%)	1.33 (0.84–2.12)	1.33 (0.83–2.12)	1.21 (0.71–2.07)
40–59 years				
Low UIC (<100)	128/1166 (10.98%)	1.32 (0.86–2.04)	1.27 (0.83–1.95)	1.40 (0.85–2.29)
Normal UIC (100–199)	95/948 (10.02%)	1.00 (reference)	1.00 (reference)	1.00 (reference)
Slightly high UIC (200–299)	37/423 (8.75%)	0.90 (0.42–1.91)	0.94 (0.44–1.99)	0.93 (0.44–1.94)
High UIC (≥ 300)	82/499 (16.43%)	1.85 (1.15–2.99) *	1.98 (1.19–3.29) *	1.90 (1.11–3.25) *
≥60 years				
Low UIC (< 100)	58/906 (6.40%)	1.04 (0.56–1.93)	1.01 (0.55–1.89)	1.19 (0.67–2.11)
Normal UIC (100–199)	64/977 (6.55%)	1.00 (reference)	1.00 (reference)	1.00 (reference)
Slightly high UIC (200–299)	42/530 (7.92%)	1.47 (0.84–2.55)	1.53 (0.86–2.71)	1.34 (0.75–2.40)
High UIC (≥ 300)	54/661 (8.17%)	1.57 (0.79–3.12)	1.63 (0.81–3.31)	1.49 (0.67–3.34)

Calculated using binary logistic regression. OR, odds ratio; CI, confidence interval. ^a^ Model 1 is the unadjusted model; ^b^ Model 2 is adjusted for sex; ^c^ Model 3 further adjusted for race, education level, annual household income, BMI, marital status, physical activity, caffeine intake, total energy intake, sodium intake, smoking status, drinking status, hypertension, diabetes, stroke history, and urinary creatinine level. * *p* < 0.05.

## Data Availability

The datasets supporting the conclusions of this article are publicly available from the NHANES (https://www.cdc.gov/nchs/nhanes/index.htm). Accessed on 26 May 2022.

## Supplementary Materials

The following supporting information can be downloaded at: https://www.mdpi.com/article/10.3390/nu14194165/s1, Table S1: The classifications of covariates; Table S2: Partial urinary iodine concentration and ORs (95% CIs) in dose-response relationship; Table S3: Weighted ORs and 95% CIs of sensitivity analysis of depressive symptoms.
