# Supplementary material for: Association of Urinary Iodine Concentration with Depressive Symptoms among Adults: NHANES 2007–2018
Source: Nutrients. 2022 Oct 7;14(19):4165. doi: 10.3390/nu14194165 (PMC9573473; doi:10.3390/nu14194165)
Supplement: Supplementary file 1 [file nutrients-14-04165-s001.zip › nutrients-1938173-supplementary.pdf]

Manuscript ID: nutrients-1938173

Title: Association of Urinary Iodine Concentration with Depressive Symptoms among Adults: NHANES 2007-2018

Table S1. The Classifications of Covariates.

Covariates	Classifications
Age (year)	20-39; 40-59; ≥ 60
Sex	Male; Female
Race	Mexican American; Other Hispanic; Non-Hispanic White; Non-Hispanic Black; Other races
Educational level	Below high school; High school; Above high school
Annual household income	< \$20000; ≥ \$20000
Body mass index	< 25 kg/m^2; 25 to < 30 kg/m^2; ≥ 30 kg/m^2
Marital status	Not living alone; Living alone
Physical activity [a]	Low; High
Smoked at least 100 cigarettes in life	Yes; No
Had at least 12 alcohol drinks/year	Yes; No
Hypertension [b]	Yes; No
Diabetes [c]	Yes; No
Ever told you had a stroke [d]	Yes; No
Caffeine intake (mg/d) [e]	Continuous
Total energy intake (kcal/d) [e]	Continuous
Sodium intake (mg/d) [e]	Continuous
Urinary creatinine level (mg/dL)	Continuous

[a] Physical activity was assessed using metabolic equivalent task (MET; expressed in minutes/week) and grouped into two groups according to the Global Physical Activity Questionnaire analysis guide.

[b] Hypertension was defined as the average systolic blood pressure ≥ 130 mmHg, or the average diastolic blood pressure ≥ 90 mmHg, or self-reported doctor-diagnosed, or anti-hypertensive medications use.

[c] Diabetes was defined as blood glycohemoglobin ≥ 6.5%, or fasting plasma glucose ≥ 126 mg/dL, or 2 h glucose (oral glucose tolerance test) ≥ 200 mg/ dL, or self-reported doctor-diagnosed, or insulin use.

[d] Stroke were defined according to a self-reported doctor-diagnosed.

[e] Caffeine intake, total energy intake and sodium intake were obtained through the 24-hour dietary recall.

Table S2. Partial Urinary Iodine Concentration and ORs (95% CIs) in Dose-response Relationship.

Urinary Iodine Concentration (μg/L)	OR	95% CI
328	1.16	(0.98 - 1.34)
342	1.19	(0.99 - 1.39)
343	1.19	(1.00 - 1.39)
359	1.23	(1.00 - 1.45)
**360**	**1.23**	**(1.01 - 1.46)**
**390**	**1.30**	**(1.02 - 1.58)**
**420**	**1.37**	**(1.03 - 1.72)**
**450**	**1.45**	**(1.03 - 1.87)**
**480**	**1.53**	**(1.03 - 2.04)**
**510**	**1.62**	**(1.02 - 2.22)**
**548**	**1.74**	**(1.01 - 2.47)**
549	1.74	(1.00 - 2.48)
566	1.80	(1.00 - 2.60)
567	1.80	(0.99 - 2.60)
582	1.85	(0.98 - 2.71)

Calculated using restricted cubic spline models. OR, odds ratio; CI, confidence interval.

The bold contents have statistically significant.

Table S3. Weighted ORs and 95% CIs of Sensitivity Analysis of Depressive Symptoms.

Urinary Iodine Concentration (μg/L)	Cases/Participants	Model 3
		OR (95% CI)
Low UIC (< 100)	237/2862 (8.28%)	1.19 (0.85-1.65)
Normal UIC (100-199)	212/2565 (8.27%)	1.00 (reference)
Slightly high UIC (200-299)	97/1141 (8.50%)	1.07 (0.71-1.60)
High UIC (≥ 300)	147/1339 (10.98%)	1.53 (1.00-2.34) *

Calculated using binary logistic regression. OR, odds ratio; CI, confidence interval.
Sensitivity analysis of further excluded participants using amiodarone, thyroid hormone replacement agents, or anti-thyroid agents and subjects with severe renal dysfunction (estimated glomerular filtration rate < 30 mL/min/1.73m^2).
Model 3 is adjusted for age, sex, race, education level, annual household income, BMI, marital status, physical activity, caffeine intake, total energy intake, sodium intake, smoking status, drinking status, hypertension, diabetes, stroke history, and urinary creatinine level. * $p < 0.05$.
